# French Adaptation of the Narcissistic Personality Inventory in a Belgian French-Speaking Sample

**DOI:** 10.3389/fpsyg.2016.01980

**Published:** 2016-12-23

**Authors:** Stéphanie Braun, Chantal Kempenaers, Paul Linkowski, Gwenolé Loas

**Affiliations:** Department of Psychiatry, Erasmus Hospital, Université Libre de BruxellesBrussels, Belgium

**Keywords:** narcissism, Narcissistic Personality Inventory, French validation, exploratory factor analysis, psychometry

## Abstract

The Narcissistic Personality Inventory (NPI) is the most widely used self-report scale to assess the construct of narcissism, especially in its grandiosity expression. Over the years, several factor models have been proposed in order to improve the understanding of the multidimensional aspect of this construct. The available data are heterogeneous, suggesting one to at least seven factors. In this study, we propose a French adaptation of the NPI submitted to a sample of Belgian French-speaking students (*n* = 942). We performed a principal component analysis on a tetrachoric correlation matrix to explore its factor structure. Unlike previous studies, our study shows that a first factor explains the largest part of the variance. Internal consistency is excellent and we reproduced the sex differences reported when using the original scale. Correlations with social desirability are taken into account in the interpretation of our results. Altogether, the results of this study support a unidimensional structure for the NPI using the total score as a self-report measure of the Narcissistic Personality Disorder in its grandiose form. Future studies including confirmatory factor analysis and gender invariance measurement are also discussed.

## Introduction

The concept of narcissism originated from Greek mythology (Hamilton, 1942) has always been of interest in psychology and psychiatry. Most researchers seem to agree with its dimensional aspect (e.g., Foster and Campbell, 2007), ranging from a normal adaptive mechanism in healthy individuals to pathological narcissism that causes distress and impairment (Ackerman et al., 2011). According to the Diagnostic and Statistical Manual of Mental Disorders [5th edition, (DSM-V); American Psychiatric Association, 2013], individuals with a Narcissistic Personality Disorder (NPD) have *significant impairments* in (1) personality functioning, either in identity (excessive reference to others for self-definition and self-esteem regulation) or in self-direction (goal-setting based on gaining approval from others; personal standards unreasonably high in order to see oneself as exceptional, or too low based on a sense of entitlement) and in (2) interpersonal functioning, either empathy or intimacy (relationships largely superficial and existing to serve self-esteem regulation). Individuals with NPD have also *pathological personality traits* such as: (1) grandiosity (feelings of entitlement; self-centeredness; firmly holding to the belief that one is better than others; condescending toward others) and (2) attention seeking (excessive attempts to attract and be the focus of the attention of others; admiration seeking). These days, two expressions of narcissism are considered: grandiosity and vulnerability (Cain et al., 2008). While grandiosity is associated with the tendency to exploit others and with the feeling of entitlement and superiority, vulnerability is related to the feeling of inadequacy and incompetence with negative affect (Maxwell et al., 2011; Miller et al., 2013).

Ideally, narcissism should be studied in its multidimensional perspective (see for example, Pincus and Lukowitsky, 2010). However, the majority of researchers tend to use the Narcissistic Personality Inventory (NPI; Raskin and Hall, 1979, 1981) to only evaluate its maladaptive grandiosity dimension. For example, Cain et al. (2008; cited by Brin, 2011) report that the NPI was used in 77% of the empirical studies dealing with the concept of narcissism. This scale is based on the description of the NPD included in the DSM-III (American Psychiatric Association, 1980), and consists of 40 forced-choice dichotomous items (between narcissistic and nonnarcissistic statements). In this scale, participants are asked to select one statement within every dichotomous item that best describes their personality or opinion. Most researchers choose this dichotomous forced-choice format whilst some others (e.g., Kubarych et al., 2004; Barelds and Dijkstra, 2010) choose to convert the forced-choice response format into a Likert one. According to Barelds and Dijkstra (2010), the correlation between the dichotomous forced-choice format and a 5-point Likert version of the scale (using the dichotomous statements as anchor points) is excellent (*r* = 0.97; *p* < 0.01). In Boldero et al. (2015) the use of a 6-point rating scale showed a similar correlation (*r* = 0.96; *p* < 0.01).

Some studies aimed to explore the multidimensional structure of the NPI using exploratory factor analysis (EFA), principal component analysis (PCA) and/or confirmatory factor analysis (CFA). Raskin and Terry's model suggests seven subscales including: Authority, Self-Sufficiency, Superiority, Vanity, Exhibitionism, Entitlement, and Exploitativeness (Raskin and Terry, 1988). Other researchers proposed alternative models, such as the 4-factor solution of Emmons (1984, 1987) that includes: Leadership/Authority, Superiority/Arrogance, Self-Absorption/Self-Admiration, and Exploitativeness/Entitlement. Kubarych et al. (2004) proposed a 2-factor or a 3-factor model with (1) Power and (2) Exhibitionism as the two factors of the first one, and (1) Power, (2) Exhibitionism, and (3) Being a Special Person as the three factors of the second one. Inconsistent results in factor structure are generally considered to be associated with methodological differences between studies (see in Boldero et al., 2015). However, even the most recent studies that have taken the current psychometric recommendations into account (performing tetrachoric correlation matrix because of the dichotomous format, using the scree plot and interpreting the items' meanings to obtain a factor solution, …), failed to replicate the 7-factor structure (Corry et al., 2008; Ackerman et al., 2011). For example, Corry et al. (2008) tested several models with confirmatory factor analysis (CFA) and concluded that a 2-factor solution (with Leadership/Authority and Exhibitionism/Entitlement as factors) appeared to be the most parsimonious model, in terms of fit indices and internal consistencies values. For Ackerman et al. (2011), these two main dimensions are useful in distinguishing the part of the adaptive content from the maladaptive content of the narcissism construct. They observed a Leadership/Authority factor similar to Corry et al.'s (2008) but suggested to split the Exhibitionism/Entitlement dimension into two parts: Grandiose Exhibitionism and Entitlement/Expoitativeness. However, Boldero et al. (2015) point toward the fact that, despite psychometrical precautions, some methodological differences remain between Corry et al.'s (2008) and Ackerman et al.'s (2011) studies regarding the criteria for the EFA (type of rotation, choice of estimator and utilization of the internal consistency indices). Moreover, Boldero et al. (2015) emphasize the superiority of the parallel analysis (Horn, 1965) over the traditional criteria (scree plot, eigenvalues) in determining the number of factors to retain, and support the choice of an oblique rotation in this context (because of probable correlated items). The results of their study showed a 2-factor structure with the binary or with the rating response's format (including a general factor and six specific factors). The authors, therefore, assumed that the data were unidimensional (Reise et al., 2010) and considered the NPI's general factor as a measure of a narcissism latent trait. They also pointed out that the rating item general factor assessed more narcissism components than the binary item.

Several NPI adaptations in various languages already exist. For example, Barelds and Dijkstra (2010) proposed a Dutch version that supports neither the 4-factor model of Emmons (1987) nor the 7-factor model of Raskin and Terry (1988). Performing an EFA, they suggested a single-factor solution or a 2-factor model explaining respectively 27.3 and 33.7% of the variance. Other adaptations in Swedish (Kansi, 2003), German (Schutz et al., 2004), and Greek (Coccosis et al., 1998) also failed to replicate the 4-factor model of Emmons (1987) or the 7-factor model of Raskin and Terry (1988).

A French adaptation of the NPI (with a 7-point Likert response format) was proposed by Brin (2011) in order to obtain the grade of Doctor in Psychology (University Laval, Québec). To our knowledge, this study has not yet been published in a peer-reviewed journal. In Brin's study, a CFA testing the 7-factor model of Raskin and Terry (1988) showed no reasonable fit indices. Multiple exploratory factors analyses (EFA) were performed with promax rotation and suggested two interesting models with two or three factors, with similar findings to Corry et al.'s (2008) regarding the first one (2-factor model called Exhibitionism/Looking for attention and Leadership/Arrogance). This study confirmed (see Raskin and Terry, 1988 for other examples) an excellent internal consistency (α = 0.91) and a gender difference where males obtained higher scores than females (as in Corry et al., 2008).

The aim of our study was to propose a French adaptation of the NPI using a sample of Belgian French-speaking students. Because our data were collected before our knowledge of the Brin's study, our own French version was used (Brin, 2011). A comparison with Brin's version showed minor differences that do not affect the meaning of the sentences. As mentioned earlier, there are large inconsistencies between studies in terms of factor structure. We therefore chose PCA as the most adequate method to explore the factor structure of this new adaptation of the scale. Regarding methodological recommendations, we expected to reproduce the results of Boldero et al. (2015) and confirm the use of a unique total score as a measure of the narcissism latent trait. We also expected to obtain similar results as in Brin (2011) in terms of internal consistency and gender differences. Because a number of researchers have highlighted and tested the possible contribution of social desirability in the responses of the NPI, we decided to explore this too and to confirm minor correlations (e.g., Auerbach, 1984; Watson et al., 1984).

## Materials and methods

### Participants

Nine hundred and forty-two students (425 males–45.1% and 517 females–54.9%) participated in this study. Participants completed a set of questionnaires including our French version of the NPI. They were in their first year in various Belgian universities (engineering–25.4%, medicine–20%, economic sciences–17.7%, sciences–11.3%, psychology–4.7% and law–2%) or college/university of applied sciences (nursing school–18.9%). Their ages ranged from 17 to 25 years (*M* = 19.55; *SD* = 1.72). Courses were given exclusively in French and participants who did not speak French since childhood were excluded from the study (this information was obtained by the demographical questionnaire).

### Measure

We used the 40-item forced-choice dichotomous version of the scale (Raskin and Hall, 1979, 1981). As described above, each item consists of a pair of narcissistic and nonnarcissistic sentences such as “I think I am a special person” and “I am not better or no worse than most people.” One point is given for each narcissistic response. Each participant completed a French version of the original English version of the NPI which had been translated in French by a Belgian psychiatrist (CD) and back translated into English by a native English speaker fluent in French. This back translation was then amended in order to be as close as possible to the meaning of the original version. The French version is provided in Table 1.

**Table 1:** **Item composition of the NPI: French translation (2016)**.

**Factor**	**N°**	**Item**
Authority	1	J'ai un talent naturel pour influencer les gens. (+) Je ne suis pas doué(e) pour influencer les gens. (−)
Exhibitionism	2	La modestie ne me correspond pas. (+) Je suis essentiellement quelqu'un de modeste. (−)
Exhibitionism	3	Je relèverais presque tous les défis. (+) J'ai tendance à être quelqu'un de plutôt prudent. (−)
Superiority	4	Je suis parfois embarrassé(e) lorsque les gens me font des compliments. (−) Je sais que je suis bon parce que tout le monde me le dit. (+)
Entitlement	5	L'idée de diriger le monde me paralyze. (−) Si je dirigeais le monde, il serait meilleur. (+)
Exploitativeness	6	Habituellement, avec mon bagou, j'arrive toujours à me tirer d'affaire. (+) J'essaye d'accepter les conséquences de mon comportement.(−)
Exhibitionism	7	Je préfère me fondre dans la foule. (−) J'aime être le centre de l'attention. (+)
Authority	8	Je réussirai. (+) Je ne me sens pas trop concerné(e) par la réussite. (−)
Superiority	9	Je ne suis ni meilleur(e) ni pire que les autres. (−) Je suis une personne hors du commun. (+)
Authority	10	Je ne suis pas sûr(e) que je pourrais être un bon chef. (−) Je me vois comme un bon chef. (+)
Authority	11	Je suis assertif(j'ai de l'assurance). (+) Je souhaite être plus assuré(e). (−)
Authority	12	J'aime avoir de l'autorité sur les autres personnes. (+) Ca ne me gêne pas d'obéir aux ordres. (−)
Exploitativeness	13	Je trouve facile de manipuler les gens. (+) Je n'aime pas quand je me surprends à manipuler les autres. (−)
Entitlement	14	Je suis intransigeant(e) quant au respect qui m'est dû. (+) On me témoigne habituellement le respect que je mérite.(−)
Vanity	15	Je n'aime pas particulièrement mettre mon corps en évidence. (−) J'aime mettre mon corps en évidence.(+)
Exploitativeness	16	Je peux lire à travers les gens comme dans un livre. (+) Les gens sont parfois difficiles à comprendre.(−)
Self-sufficiency	17	Si je me sens compétent(e), je suis disposé(e) à prendre la responsabilité de prendre les décisions. (−) J'aime prendre la responsabilité de prendre des décisions. (+)
Entitlement	18	Je souhaite être raisonnablement heureux(se). (−) Je désire être quelqu'un aux yeux du monde. (+)
Vanity	19	Mon corps n'est pas spécialement beau. (−) J'aime regarder mon corps. (+)
Exhibitionism	20	J'essaye de ne pas me faire valoir aux yeux d'autrui. (−) Je sais me mettre en avantage si j'en ai l'opportunité. (+)
Self-sufficiency	21	Je sais toujours ce que je suis en train de faire. (+) Parfois, je ne suis pas sûr(e) de ce que je fais. (−)
Self-sufficiency	22	Je dépends parfois d'autres personnes pour que les choses soient faites. (−) Je dépends rarement de quelqu'un d'autre pour que les choses soient faites. (+)
Exploitativeness	23	Parfois, je raconte de bonnes histoires. (−) Tout le monde aime bien écouter mes histoires. (+)
Entitlement	24	J'attends beaucoup des autres. (+) J'aime faire des choses pour les autres personnes. (−)
Entitlement	25	Je ne serai jamais satisfait(e) avant d'avoir tout ce que je mérite. (+) Je prends les choses agréables comme elles viennent. (−)
Superiority	26	Les compliments m'embarrassent. (−) J'aime être complimenté(e). (+)
Entitlement	27	J'ai un fort désir de pouvoir. (+) Le pouvoir, en ce qui me concerne, ne m'intéresse pas. (−)
Exhibitionism	28	Je ne me préoccupe pas des nouvelles modes. (−) J'aime lancer de nouvelles modes. (+)
Vanity	29	J'aime me regarder dans un miroir. (+) Je ne me sens pas particulièrement attiré(e) par le fait de me regarder dans un miroir. (−)
Exhibitionism	30	J'apprécie réellement être le centre de l'attention. (+) Cela me met mal à l'aise d'être le centre de l'attention. (−)
Self-sufficiency	31	Je peux faire de ma vie ce que je veux. (+) Les gens ne peuvent pas toujours vivre leur vie comme ils le veulent. (−)
Authority	32	Etre reconnu(e) en tant qu'autorité ne signifie pas grand-chose pour moi. (−) Les gens semblent toujours reconnaître mon autorité. (+)
Authority	33	Je préférerais être un chef. (+) Cela fait peu de différences pour moi d'être un chef ou non. (−)
Self-sufficiency	34	Je serai une personne formidable. (+) J'espère que je vais être couronné(e) de succès. (−)
Exploitativeness	35	Parfois, les gens croient ce que je leur raconte. (−) Je peux faire croire n'importe quoi à n'importe qui. (+)
Authority	36	Je suis un chef né. (+) Etre un ≪ Homme de tête ≫ est une qualité que l'on met longtemps à acquérir. (−)
Superiority	37	Je souhaite que quelqu'un écrive un jour ma biographie. (+) Je n'aime pas que les gens se mêlent de ma vie privée pour quelques raisons que ce soient. (−)
Exhibitionism	38	Je suis vexé(e) lorsque les gens ne remarquent pas mon allure vestimentaire quand je sors. (+) Cela ne me gêne pas d'être fondu(e) dans la foule lorsque je sors. (−)
Self-sufficiency	39	J'ai plus de capacités que les autres personnes. (+) Je peux apprendre beaucoup des autres personnes. (−)
Superiority	40	Je suis plus comme tout le monde. (−) Je suis une personne extraordinaire. (+)

*(+) Narcissistic responses; (−) Non-narcissistic responses*.

The NPI was administered with other self-report scales including a social desirability measure (Crowne and Marlowe, 1960). This scale consists of 33 forced-choice items in which participants have to decide whether they agree or not with sentences supposed to be highly socially desirable. The higher the total score, the higher the social desirability's influence in the subjects' responses. Cronbach's α coefficient of the Marlowe-Crowne scale is acceptable (α = 0.73) but not very high. Some demographical information (such as civil status, native language, medical data, academic courses …) was collected in the latter part of the session.

### Procedure

The assessment was approximately 1 h long and took place during a scheduled class time. The students were informed that the study received the approval from the Ethical Committee of the Erasmus Hospital. Participation was voluntary and anonymous. Participants were informed that feedback would be available at a later stage by contacting the authors of the study. They responded to the questionnaires on answer sheets in a semi-random order to avoid any bias linked to the presentation sequence. No compensation was offered for their participation.

### Analysis

We conducted an exploratory principal component analysis (PCA) on the unrotated tetrachoric correlation matrix of the NPI 40 items. This method was chosen because of the binary format of the items and according to several recommendations for factor analyses in dichotomous variables (Carroll, 1961; Muthén, 1978; Muthén and Hofacker, 1988; Kubinger, 2003). According to Kubinger (2003), tetrachoric correlations would lead to more content valid results in case of dichotomous variables because factor analysis is based on Pearson correlations which require interval scaled variables. Indeed, simulation studies (using EFA or CFA) have recently shown that the solutions obtained with polychoric correlations provide a more accurate reproduction of the measurement model used to generate the data (Holgado-Tello et al., 2010).

In addition to an interpretation of the factors content, we used the following indices to determine the number of factors: the Kaiser criteria (Kaiser, 1960), the scree test (Cattel, 1966) and the parallel analysis (Horn, 1965). This last procedure is a Monte Carlo simulation method known to be more efficient than the Kaiser criteria (eigenvalue > 1) and the scree test in determining the number of factors to retain. Reliability (or internal consistency) was estimated by Cronbach's α coefficient (Cronbach, 1951).

Student's *t*-test was performed to investigate gender differences in the NPI total score and Pearson correlations were then applied between the NPI total score and the Crowne-Marlowe total score in order to explore the contribution of social desirability.

Statistical analyses were performed with Statistica 7.1 (Statsoft Inc., 2005^1^) and SPSS 22.0 (IBM Corp. Released, 2013).

## Results

### Principal components analysis and reliability

**Figure 1:**
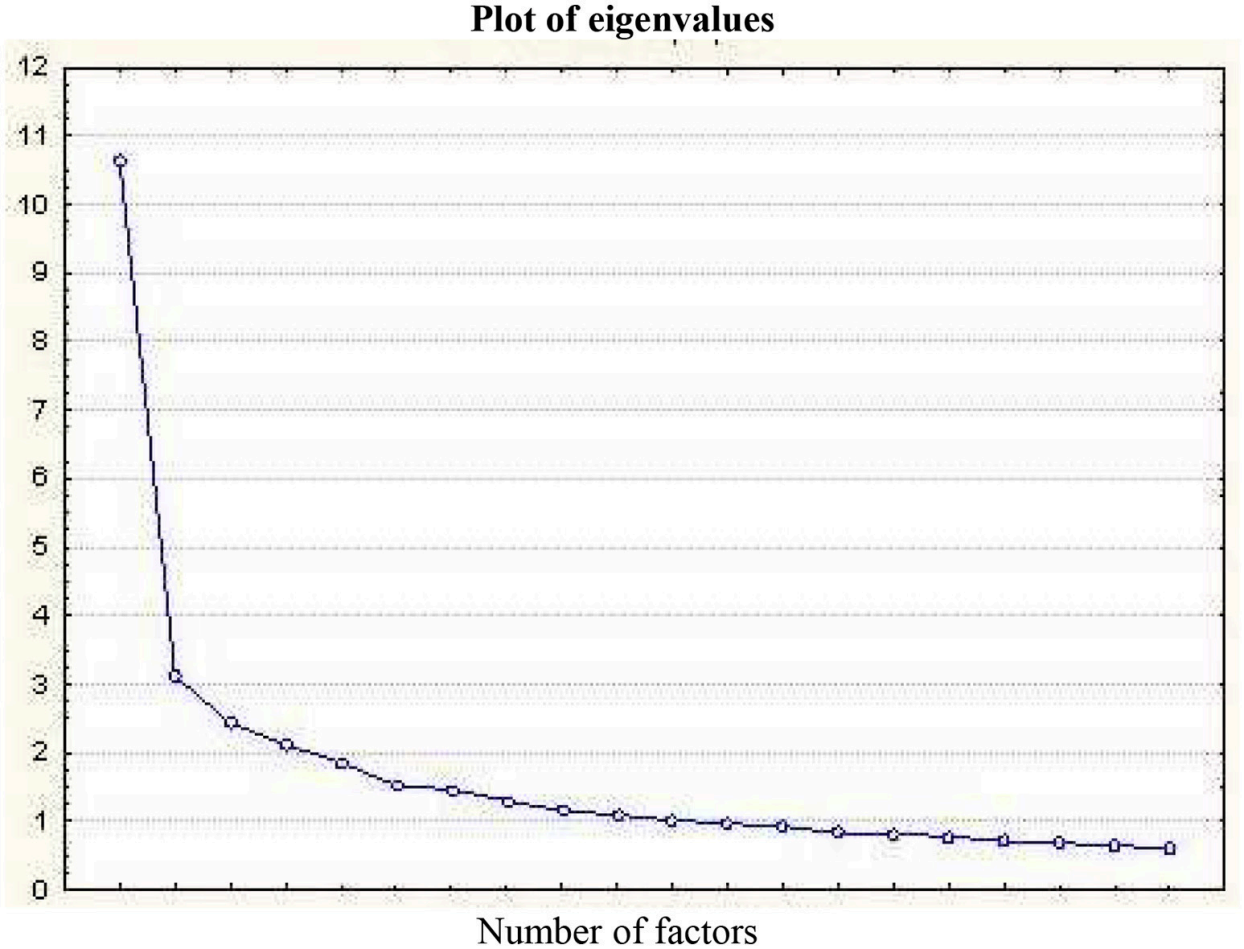
**Plot of eigenvalues**.

In agreement with the Kaiser criteria, the PCA results indicate an 11-factor solution with an eigenvalue greater than 1, representing together 69.28% of the variance. The scree test (see Figure 1) showed a clear inflection after the first factor, which explains 26.59% of the variance (with an eigenvalue of 10.63). The parallel analysis suggests retaining 7 factors (with a simulated eigenvalue of 1.41 and a real eigenvalue of 1.30 for the 8th factor). Together, these results suggest 7 dimensions for the NPI (57.87% of variance explained) but with a first factor explaining the major part of the variance. The unrotated tetrachoric correlation matrix of factor loadings is provided in Table 2. Taking into account the loadings greater than 0.40, the first factor contains 20 items without any significant crossloadings. We found many crossloadings between several items that load on the 6 remaining factors, some corresponding to a combination of items from the original Raskin and Terry study (Raskin and Terry, 1988, F2: items 15, 19, 26, 28, 29, 33, and 38 from the Vanity, Superiority, Authority and Exhibitionism factors; F3: items 8, 11, 24, and 25 from the Authority and Entitlement factors; F4: items 9, 31, 34, 39 from the Superiority and Self-Sufficiency factors; F5: items 6 and 8 from the Authority and Exploitativeness factors; F6: items 4 and 26 from the Superiority factor; and F7: item 28 from the Exhibitionism factor). Finally, Cronbach's α coefficient of the total score suggests satisfactory reliability (α = 0.92, with an average interitem tetrachoric correlation of 0.23).

**Table 2:** **Unrotated tetrachoric correlation matrix of factor loadings**.

**Items**	**F1**	**F2**	**F3**	**F4**	**F5**	**F6**	**F7**
1	**−0.58**	0.23	−0.26		0.36		
2	−0.38				0.25		
3	**−0.47**					0.30	0.30
4	**−0.49**	−0.27				**−0.72**	
5	**−0.56**	0.29					0.26
6	**−0.46**	0.20			**0.50**	0.23	
7	**−0.66**	−0.25		0.30	0.29		
8	−0.34		**−0.45**		**−0.40**	0.24	
9	**−0.66**	−0.21		**−0.45**			
10	**−0.67**	0.33			−0.20		
11	**−0.50**	0.22	**−0.48**				
12	**−0.59**	0.23		0.32			
13	**−0.62**	0.26			0.24		
14	−0.22		0.26				−0.31
15	**−0.53**	**−0.44**			−0.20	0.21	
16	**−0.41**			−0.29	0.33		
17	**−0.43**			0.23			−0.35
18	**−0.51**						
19	**−0.51**	**−0.40**	−0.25		−0.36		
20	**−0.50**	−0.28		0.25			
21	−0.25	0.34	−0.30	−0.34		−0.29	
22		0.33	−0.38				−0.30
23	**−0.51**		−0.34		0.25		−0.38
24			**0.65**	−0.20			
25	−0.39		**0.59**				
26	−0.33	**−0.41**	−0.23			**−0.53**	0.27
27	**−0.74**	0.39	0.23	0.23			
28	−0.38	**−0.41**		0.27			**−0.41**
29	−0.35	**−0.57**			−0.29	0.25	
30	**−0.73**			0.29	0.28		0.21
31	−0.38		−0.20	**−0.46**			
32	**−0.65**	0.27					
33	**−0.68**	**0.42**	0.22	0.29	−0.29		
34	−0.21	−0.24	−0.27	**−0.42**			−0.23
35	**−0.51**	0.21			0.39		
36	**−0.81**	0.25					
37	**−0.45**	−0.34					
38	**−0.55**	**−0.46**					−0.39
39	**−0.62**		0.40	**−0.52**			
40	**−0.65**	−0.28		−0.36			

*Criterion for significance is fixed to 0.40 (boldface)*.

*F1, main factor; F2, combination of Vanity, Superiority, Authority and Exhibitionism factors; F3, combination of Authority and Entitlement factors; F4, combination of Superiority and Self-Sufficiency factors; F5, combination of Authority and Exploitativeness factors; F6, Superiority; F7, Exhibitionism*.

### Gender difference and social desirability

Similarly to Brin (2011), we found a significantly higher total score for males than for females: *Mf* = 12.30, *SDf* = 6.09, vs. *Mm* = 14.40, *SDm* = 6.64, *t*_(940)_ = −5.17, *p* < 0.001. Correlations between NPI scores and social desirability are also statistically significant for the total group and for females (*r* = −0.119, *p* < 0.01 for the total group; *r* = −0.052, *p* = 0.322 for males; *r* = −0.182, *p* < 0.01 for females). However, these negative correlations should be interpreted with caution because they are rather small (< 0.30) and suggest weak associations between the two scales.

## Discussion

In this study, we proposed a French adaptation of the NPI in a French-speaking Belgian subjects sample with the aim to explore its psychometrical aspects. Given the inconsistencies of previous studies (e.g., Corry et al., 2008; Ackerman et al., 2011) and considering methodological recommendations (see on Boldero et al., 2015 for a review), we performed a PCA on an unrotated tetrachoric correlation matrix and used parallel analysis to interpret our results. Just like Boldero et al. (2015), we observed that the first of the 7 factors we obtained explains the largest part of the variance. We therefore agree with them and other researchers such as Barelds and Dijkstra (2010) who consider the total score of the NPI as a good measure of the narcissistic trait at least in its grandiosity aspect.

Some psychometrical methods support the unidimensional hypothesis. Although Reckase (1979) proposes to retain a rate of variance explained by the first factor higher than 20% (which is the case in our study), Carmines and Zeller (1979) suggest four criteria to apply on the unrotated matrix: (1) the first component should explain a large part of the variance (>40%), (2) the next components should explain fairly equal components of the remaining variance, (3) all or most of the items should have substantial loadings on the first factor (>0.30), (4) all or most of the items should have their highest loadings on the first factor. Three of the four criteria were met in our study: (1) the part of the variance explained by the first factor is 26.59%, which corresponds to nearly half (46%) of the total variance explained (57.87%) (2nd criteria), (2) 35 items load on the first factor above 0.30 (3rd criteria), and (3) 28 items that met the 4th criteria (see on Table 2). The 1st criteria—the strictest—is not met. Finally, Lord (1980) envisages a procedure which implies creating an index (1st eigenvalue—2nd eigenvalue/2nd eigenvalue—3rd eigenvalue). In our study, this index was (10.63–3.11)/(3.11–2.43) = 11.05. Unfortunately, the drawback of this method is the absence of consensus for determining a cut-off score to identify unidimensionality.

Because our data collection took place some years ago, we did not convert the forced-choice response format into a Likert format scale, as suggested by Boldero et al. (2015). This could be considered a limitation in our study. A comparison of the two response formats in Boldero et al. (2015) suggested that a rating format enhances the information obtained with the NPI. Moreover, Boldero et al. (2015) express concerns regarding the influence of social desirability in the dichotomous format. They hypothesized that several narcissistic vs. nonnarcissistic statements are not equivalent in terms of social desirability and that this could underestimate the ranking of the subjects on the narcissism latent trait. In our study, we found statistically significant correlations with social desirability but these were inferior to 0.30. Interestingly, the correlation is significant for females but not for males. This might indicate a potential instability of the measure across gender, at least with the binary format. Moreover, we believe that it could be difficult for participants to choose a “right” answer in a forced-choice situation. Sometimes participants find themselves having to choose between two unsatisfactory answers. This is especially problematic (in terms of methodology) when the range between the items is variable. While some pairs are clearly antagonistic (“I have a natural talent for influencing people” vs. “I am not good at influencing people”), we doubt that it is the case for other pairs (“I find it easy to manipulate people” vs. “I don't like it when I find myself manipulating people”).

Regarding gender differences, we found a higher NPI total score for males than for females, thus replicating other studies' findings (Corry et al., 2008; Brin, 2011). However, we did not test metric gender invariance; results should therefore be interpreted with caution. Indeed, testing for gender invariance could ensure that the measure is not influenced by systematic response's bias linked to gender (Meredith, 1993). Significant correlations we observed between the NPI's total score and social desirability in females but not in males might be another argument in favor of testing gender invariance in a future study. If this invariance condition was accepted, the difference on the NPI's total score we found between males and females could be interpreted in terms of stereotypes in gender roles. Considering the environmental aspects of this hypothesis (education, culture …), it would be interesting to develop a version of the NPI specifically aimed at children and test a possible interaction between age and gender.

In conclusion, although Narcissism Personality Disorder (NPD) is considered a multidimensional construct in the DSM-V, results from our research study support the idea that the NPI cannot explore its entire complexity. The lack of consensus in literature regarding the limits between normal and pathological narcissism renders its evaluation particularly complicated (Pincus and Lukowitsky, 2010) and future studies are necessary. Vulnerability aspects of the NPD should be explored further and integrated in new measures such as in the Pathological Narcissism Inventory (PNI; Pincus et al., 2009). Notwithstanding, the general limitations of self-report scales, the use of the NPI and the interpretation of its total score may be useful for clinical purposes. Our French adaptation showed good psychometrical properties, congruent with the literature data which allows us to consider it a satisfactory instrument to measure narcissism in its grandiosity dimension. For future studies, we suggest using a rating scale rather than the dichotomous format and to test the unidimensional hypothesis with CFA. A gender invariance measure should also be performed to confirm the stability of the scale across gender and to clarify the social desirability influence.

## Ethics statement

The study received the approval from the Ethical Committee of the Erasmus Hospital. The study took place during a scheduled class time.

## Author contributions

PL initiated and designed the study and provided the funding, SB and CK collected the data, SB and GL co-wrote the paper.

## Funding

The present study was supported by the Belgian National Funds for Scientific Research (grants 1.5.123.04, 1.5.175.06, and 3.4.553.01.F).

### Conflict of interest statement

The authors declare that the research was conducted in the absence of any commercial or financial relationships that could be construed as a potential conflict of interest.

## Footnotes

^1^Statsoft Inc., Tulsa, OK, USA, 2005.
